# P-2065. Profile and Risk Factors of Breakthrough COVID-19 Infections Among Healthcare Workers Post-Vaccination

**DOI:** 10.1093/ofid/ofae631.2221

**Published:** 2025-01-29

**Authors:** Mario M Panaligan

**Affiliations:** UERMMMCI, Taguig, National Capital Region, Philippines

## Abstract

**Background:**

This study aimed to assess breakthrough COVID-19 infections in vaccinated healthcare workers, characterize their demographic and clinical profiles and identify associated risk factors.

**Methods:**

A cross-sectional study was conducted in four hospitals using a stratified sampling approach. Data were collected via standardized questionnaires and analyzed using descriptive statistics for demographic and clinical features, with Chi-square or Fisher’s exact test to examine for significant associations and T-tests or Mann-Whitney U Tests for significant differences.

**Results:**

Among 378 vaccinated healthcare workers, all experienced breakthrough infections, the majority during the surge of the omicron variant. Overall, 60% were females, and 91.5% were below 44. There were 172 (45.5%) nurses, 66 (17.5%) physicians, and the remaining were paramedical personnel. Breakthrough infections were shorter in vaccinated individuals (mean 11 days, SD = 5.58) compared to unvaccinated (mean 14 days, SD = 8.11). For those who developed their first breakthrough infection, the need for hospitalization and abnormal clinical and laboratory findings were significantly lower compared to unvaccinated. Among the vaccinated, higher rates of first breakthrough infection were significantly associated with being healthcare-associated and part of an outbreak. During the surge of infections due to the omicron variant, community-associated exposure and hospitalization were significantly higher among unvaccinated patients. Healthcare-associated contact was significantly associated with vaccinated people. By regression analysis, breakthrough infections due to omicron were significantly associated with a higher number of vaccine doses (OR 4.465 95% CI 1.978, 10.083) and hospitalization (OR 11.2 95% CI 2.386, 52.728). The probability of contracting the omicron variant with unknown exposure in patient care units within the past 14 days is lower (OR 0.389, 95% CI 0.174-0.866).

**Conclusion:**

Breakthrough COVID-19 infections occurred in all vaccinated healthcare personnel, with new variants and healthcare-related duties playing significant roles.

**Disclosures:**

All Authors: No reported disclosures

